# Innovation and valuation of Chinese born-global firms

**DOI:** 10.1371/journal.pone.0325214

**Published:** 2025-06-27

**Authors:** Ruize Cai, Chenyu Zhang, Yiming Xie, Minho Kim

**Affiliations:** 1 Department of International Trade, Jeonbuk National University, Jeonju, South Korea; 2 Business School, Xijing University, Xi’an, China; Al Ain University, UNITED ARAB EMIRATES

## Abstract

With the advancement of corporate globalization, an increasing number of small and medium-sized enterprises (SMEs) have leveraged globalized resources to achieve accelerated growth mode that significantly depart from the traditional gradual development trajectories of large enterprises. Notably, the emergence and evolution of born-global (BG) firms have attracted substantial scholarly attention in international business research. This paper studies the innovation and valuation of Chinese born-global (BG) firms, based on dynamic capabilities theory and resource-based view, explores the determinant factors of becoming BG firms, and explores an empirical analysis of changes in the value of BG firms. This paper utilizes the OLS model and panel model, as well as Heckman two-stage, propensity score matching (PSM), and heterogeneity analyzes. We conducted some empirical tests on financial data from 2007 to 2021. The empirical results show that the implementation of the BG mode by enterprises contributes to the growth of corporate value and innovation plays a positive moderating role in this relationship. In addition, the determinant factors for a company to adopt the BG mode are total assets, ownership, and the rate of the largest shareholder. Heterogeneity analysis indicates greater impact on private, foreign, and eastern regional firms. The Heckman two-stage selection model effectively addressed the identification requirements for exclusion restriction variables, while the PSM methodology demonstrated improved covariate balance distributions across matched groups. This dual approach collectively mitigated endogeneity concerns and enhanced the robustness of estimation outcomes. Finally, this study provides business managers with a valuation model for enterprise internationalization, which helps small and medium-sized enterprises choose BG mode to start the internationalization process in their initial stage. Furthermore, this study has significantly enriched the existing literature concerning innovation, corporate value, and equity characteristics of BG firms, while establishing novel theoretical perspectives and methodological avenues for subsequent research investigations.

## 1. Introduction

In recent years, the internationalization of enterprises has gradually expanded, and the internationalization strategies of large enterprises have gradually been applied to the development of small and medium-sized enterprises (SMEs). Some SMEs have gradually become outstanding international enterprises with market share and voice in their field by virtue of their technological innovation and organizational flexibility [1,2]. Such SMEs usually carry out international business in the initial stage and they are called born-global (BG) firms [3,4]. The establishment and operation of BG firms is becoming a popular business mode and attracts widespread attention and discussion in academic circles [5,6]. Trudgen and Freeman [7] examined performance depending on the characteristics and strategies of BG firms. Knight and Cavusgil [8] proposed that innovation culture, knowledge, and capabilities are the key factors of BG firms, and summarized the key topic of the development of BG firms in the past ten years [9]. By utilizing interviews and questionnaires, Gerschewski, Rose [10] found that the key drivers of BG firm performance are international entrepreneurial orientation, focus on product/service quality, and competitor orientation. Chen [11]  also employed a questionnaire survey method, utilizing domestic and international market conditions, manager characteristics, and organizational orientation to construct a comprehensive framework, exploring the determinants of the formation of born global firms. Due to the delayed development of China’s economy, the internationalization process of Chinese enterprises is very slow. The small number of BG firms in China has resulted in less quantitative research on Chinese BG firms. Therefore, the phenomenon of BG has not attracted the attention of Chinese scholars. The effects of BG mode on corporate value, particularly in Chinese setting, have not reached to consensus and still lack the theoretical explanation. Moreover, due to the lack of early financial data and equity structure, especially the role of BG firms’ equity characteristics in firm formation remains to be studied. BG research also has some limitations and the development strategy of Chinese enterprises under the BG mode needs further discussion.

The development patterns of BG firms in developed countries have been extensively studied. However, the results vary slightly due to the different countries and analysis methods employed. For example, recent research shows that BG firms are mainly high-tech companies, but we found that BG companies also exist in traditional export industries, such as clothing, electrical appliances, and plastic products [12–14]. The traditional view is that BG firms are generally only born in countries with small domestic market demand [15–17]. This view is currently being challenged. Kim, Lee [18] found that South Korean manufacturing BG firms can effectively utilize assets and equity, and have better growth potential. Ferguson, Henrekson [19] found no evidence that Swedish companies have advantages in value growth by choosing the BG mode, which is inconsistent with other studies. The influence of entrepreneurs’ personal characteristics and environmental factors has been comprehensively analyzed on BG firms in European and Latin American countries [20]. Based on data from small and medium-sized exporting firms in Norway, Denmark, and France, Moen, Falahat [6] found that the degree of internationalization of a firm largely depends on its behavior shortly after establishment. Andersson, Danilovic [21] utilized literature review and interviews to compare the differences in the internationalization process of “born global” firms between China and the West, analyzing the factors for success from entrepreneurial, organizational, strategic, and external environmental perspectives. It was also pointed out that Chinese research pays less attention to organizational factors, placing greater emphasis on the influence of government policies, geographical location, and relational networks. Zhang, Kim [22] found that the ownership and size differences of Chinese BG firms have significant effects on financial performance. However, in the context of the gradual internationalization of Chinese enterprises, SMEs also face opportunities and challenges. SMEs choose the BG mode in the start-up stage and the impact of this on corporate value remains to be explored. In the process of enterprises adopting the BG mode, the mechanisms influencing innovation and determinant factors have not yet been fully explained. This study distinguishes itself from prior investigations by departing from traditional data collection modalities (surveys, literature reviews, or interviews) and instead presents quantitative empirical findings through systematic integration of multi-source data on Chinese small and medium enterprises’ adoption of BG mode. Therefore, we propose three questions to address this research gap.

(1) Whether the BG mode helps enhance corporate value.
(2) How innovation affects the BG mode and corporate value?
(3) What are the determinant factors for becoming a BG firm?

Building upon the above issues, this study employs a systematic approach combining theoretical framework development with comprehensive literature review. Through rigorous empirical analysis of the BG mode and innovation of Chinese SMEs utilizing the China Stock Market and Accounting Research (CSMAR) database, we also identify the key determinants factors of becoming a BG firm. First, we analyze the impact of the BG mode on corporate value. Secondly, we propose the possible moderating effect of innovation on the relationship between the BG mode and corporate value. Then, we further verify the determinant factors that affect the choice of the BG mode, referring to assets and equity. To test our hypotheses, we employ multiple methods, including the OLS model, panel model, Heckman two-stage, propensity score matching (PSM) model analysis, and heterogeneity analysis. We believe that through the above process, we prove that the BG mode has a significant positive effect on corporate value and innovation is one of the moderating mechanisms of the BG mode. At the same time, we also prove that total assets, ownership, and the rate of the largest shareholder are the determinant factors affecting BG firms. The adoption of the BG mode by private-owned enterprises (POEs), foreign-owned enterprises (FOEs), or firms located in the eastern region has a significant effect on corporate value. This study helps to improve the development strategy of BG firms and encourages SMEs to choose to develop the BG mode.

We make three major contributions to the literature. First, we confirm that when firms select BG mode in China, corporate value increases. Although previous studies provided views on the influence of the BG mode in China, there is still a lack of consensus on the positive effect of the BG mode on corporate value and exploring the impact mechanism of this phenomenon. Therefore, this study investigates the mechanisms through which firms’ continuous innovation sustains competitive advantage within from the perspective of dynamic capability. The study found that the positive effect of the BG mode on corporate value is likely to increase with innovation. Innovation is considered one of the main strategies for corporate growth and Chinese companies invest a lot of money in the field of research and development. Therefore, our research results show that innovation is the most important strategic resource for enterprises and a source of competitive advantage. Chinese start-ups insist on independent innovation, which helps them open up to the international market. We emphasize that innovation will bring huge competitive advantages to enterprises and promote the improvement of corporate value.

Secondly, this paper also provides solutions for the determinant factors of the BG mode based on resource-based view. We propose three main factors that influence firms entering the BG mode: the total assets, ownership, and the rate of the largest shareholder. After analyzing the fixed and random effect models, we examined the specific characteristics of BG firms, especially SMEs in China. From a micro perspective, we further interpreted how the BG mode affects the development of firms. By observing micro variables, we find which of the corporate determinant factors are the main obstacles to improvements of corporate value and then help managers judge whether firms select the BG mode or when to enter the BG mode. Based on the heterogeneity analysis of ownership, we found that POEs are more suitable for developing the BG mode, compared to state-owned enterprises (SOEs) and FOEs. In terms of regional division, if firms of the eastern region develop the BG mode, the probability of corporate value growth is higher.

Third, the study presents inferences that have been tested by the authors for validity and robustness. First, we employed a dummy variable and developed a corporate valuation model with the BG mode. This makes the evaluation of Chinese firms on the BG mode more comprehensive. We used a pooled OLS regression method to verify the positive effect of the BG mode on corporate value and used the panel regression method to make the results more robust. We also analyze the relationship between the BG mode and unique characteristics of firms using a logistics model, emphasizing that the characteristics of firms are affected by the BG mode. In order to further test the robustness of Hypotheses 1 and 2, we also used Heckman two-stage and PSM to avoid endogeneity problems such as sample selection bias through randomized controlled trials.

The remainder of the paper is organized as follows. In the next section, we construct the theoretical framework, review the relevant literature, and propose three hypotheses for empirical studies. Section 3 presents the data and the main variables. In Section 4, we describe the methodology and explain the regression model specification. In Section 5, we report empirical results, including descriptive statistics, correlation results, and the regression results on the BG mode and corporate value. We also developed the results for other tests to interpret the possible causal relation. In Section 6, we provide discussion and implications, and summarize the conclusions.

## 2. Related theory, literature review, and research hypotheses

### 2.1. Related theory

#### 2.1.1. Dynamic capabilities theory and the moderating role of innovation.

Dynamic capabilities theory originated from the characteristics of the changing market environment in the 1990s [23]. The increasingly dynamic market environment, the accelerated pace of technological innovation, the internationalization of the economy and the globalization of the market, and the diversification of customer needs have resulted in faster and faster competition content and lower sustainability of competitive advantages. Only by continuous innovation can firms achieve sustained success. Dynamic capabilities theory provides a compelling theoretical basis for studying how innovation moderates the relationship between BG mode and corporate value. This view emphasizes that BG firms, characterized by their rapid internationalization from inception, must develop and reconfigure their innovation capabilities bases to maintain competitive advantage in dynamic global markets [8,24]. As Zahra, Sapienza [25] interpreted, dynamic capabilities enable BG firms to sense and seize international opportunities while transforming and updating their knowledge base to remain competitive. The intensity and nature of innovation activities significantly affect how these capabilities are transformed into corporate value.

In this theoretical framework, several key terms warrant closer examination. First, the concept of “absorptive capacity” plays a pivotal role in explaining how BG firms exploit innovation [26]. These firms must not only develop new technology, but also effectively absorb and apply external technology from different international markets. Second, “ambidexterity” becomes crucial in balancing exploration and exploitation activities, especially for BG firms that operate in multiple markets simultaneously [27]. The theoretical framework suggests that innovation strengthens the relationship between BG mode and corporate value through three main mechanisms:

(1) Market adaptation: Innovation capabilities enable rapid customization of products for different international markets [24].
(2) Knowledge integration: Innovation facilitates the synthesis of cross-border knowledge flows to create unique value propositions [8].
(3) Risk mitigation: Continuous innovation helps BG firms cope with the uncertainty inherent in international markets [28,29].

However, this relationship is not uniformly positive. Excessive innovation by the firms can lead to resource fragmentation and coordination challenges potentially undermining the effectiveness of the strategy [30]. This indicates that innovation, as a moderating role, may lead to an uncertain relationship between BG mode and corporate value. The proposed theoretical framework integrates these insights, positioning innovation as both an enabler and a potential constraint in the relationship between BG mode and corporate value. It highlights the uncertainty of this relationship, suggesting that the optimal level and type of innovation depends on factors such as industry dynamics, market diversity, and the firm’s stage of BG development.

#### 2.1.2. Resource-based view on driving forces of born global formation.

The resource-based view provides a powerful theoretical perspective for studying how firm-internal factors, especially financial status and ownership structure, affect the choice of BG mode of SMEs. This theory emphasizes that firms are a collection of resources and capabilities, and their strategic decisions are fundamentally influenced by their unique resource endowments.

Financial status constitutes a key resource dimension that directly affects the ability of SMEs to expand internationally. As Gleason, Madura [31] demonstrated, SMEs with stronger financial status are more capable of implementing BG mode. In contrast, financially constrained firms may choose less resource-demanding entry other modes, which require lower capital investment but have limited market control and growth potential [32,33].

The ownership type of the firm significantly affects the choice of the BG mode through different resource allocations and institutional legitimacy considerations. SOEs often benefit from strong government support and resource advantages, enabling them to make large-scale, politically driven international investments [34]. In contrast, as Delios, Zhou [35], Zhang and Dai [36] points out, POEs generally adopt more market-oriented approaches, focusing on efficiency-seeking strategies and gradual international expansion. This divergence stems from differences in resource dependence, with SOEs relying more on political networks, while POEs emphasize market capabilities [37]. In addition, ownership type also affects risk propensity, with SOEs typically taking on riskier projects due to implicit government guarantees, while POEs have a more cautious and profit-oriented expansion model [38]. However, FOEs often exhibit unique corporate strategic behaviors due to their unique resource allocation and institutional embeddedness [39]. These enterprises often leverage their parent company’s global network and advanced technological capabilities to implement complex internationalization strategies [40]. Unlike SOEs and POEs, FOEs often adopt a global integration strategy that emphasizes efficiency and standardization across markets [41]. Nonetheless, FOEs face unique challenges in navigating the institutional environment of host countries, especially in emerging markets, which may affect their strategic choices and market entry modes [42]. The three distinctions of SOEs, POEs, and FOEs provide a more comprehensive framework for understanding the BG mode driven by ownership type.

Ownership structure represents another pivotal internal factor influencing BG mode, which also affects resource allocation and strategic decision-making processes. Some studies found that companies with concentrated ownership, especially those dominated by institutional investors or founding families, tend to show more risk aversion in their international expansion strategies [43,44]. In contrast, companies with dispersed ownership structures may show greater active globalization tendencies, as the dispersion of ownership reduces individual risk exposure [45].

The interaction between financial status and ownership structure creates unique strategic paths for BG mode. The resource-based view emphasizes the importance of complementarity between financial status and ownership structure in shaping the choice of BG mode. As Peng [46] interpreted in his institution-based view of international business strategy, firms that effectively combine financial capabilities with governance structures can develop unique resource configurations that support their sustained competitive advantage in international markets.

### 2.2. Literature review

Whether the BG mode influences the corporate value is gradually becoming a topic of concern. Current studies have mainly analyzed BG firms in developed countries and obtained different results. Some studies suggest that the BG mode tends to result in positive financial performance [47]. The BG mode is positively related to the firm’s financial performance, especially for SMEs, but it has nothing to do with the firm’s size [48]. Furthermore, the innovation also helps start-ups gain a competitive edge and quickly take market share [49]. However, other studies suggest that enterprises that choose the BG mode do not necessarily lead to higher firm growth [19]. R&D investment can cause high levels of transaction costs that may be an obstacle to accessing debt financing [50] and the corporate value will be influenced. In recent years, with the development of the internet, the digitization of enterprises helps enterprises to expand overseas markets, which has also provided a technical basis and possibility for innovation. According to the literature analysis, enterprise digitization also helps the improvement of corporate value. Strandberg [51] identified external and internal background factors that influence internationalization behavior for digital BG by case studies. Mingione and Abratt [52] also analyzed the elements of born-digital startups to become competitive corporate brands in the context of the digital age.

Additionally, most scholars believe that the driving factors of BG firms’ growth are related to enterprises, entrepreneurship, organizational learning, enterprise network, and financial conditions [48,49,53,54]. Entrepreneurship is one of the key factors to determine the choice of the BG mode. Cao and Ma [55] studied the roles of business, network, and entrepreneur-specific factors in promoting the rapid internationalization of BG companies, which all have a positive impact on the rapid internationalization of BG companies. In analyzing Brazilian BG companies, Stocker, Abib [56] found that internationalization was driven by the background and international experience of entrepreneurs, who followed different expansion strategies in the international market. In analyzing 13 countries in Europe and Latin America, both the personal characteristics of entrepreneurs and environmental factors have strong impacts on the formation of BG companies [20]. Moreover, organizational learning and enterprise networks will also influence start-ups to become BG enterprises. For example, influencing factors of BG mode strategy selection may be the degree of internet capability and entrepreneurial orientation, which constitute the framework of the internationalization process of a BG [57]. From the two dimensions of digitalization degree and internationalization, it was found that the emergence of BG digital enterprises presents a unique phenomenon of international enterprises. The digitalization of value chain activities is not closely related to the internationalization dimension of native-digital companies, but the business model will affect the internationalization of native-digital companies [58]. Lee, Park [59] found that South Korean start-ups, entrepreneurships, operation systems, and support systems had positive impacts on corporate value, but the entrepreneurial spirit and operation system had no significant impact on corporate value growth and value creation had no significant mediating effect on global growth.

There are also some related research methods involving BG firms. Some scholars also adjust the measurement of the BG mode to 1 or 6 years [18], the volume of exports from 25% to 50%, and control the number of employees [19]. Then, Heckman two-stage and PSM quantitative analyzes are performed to further verify the experimental results and support the above hypotheses [60]. Some studies have also compared the difference in sales, employment, and value added by firm age for BG versus other exporting firms [19].

### 2.3. Research hypotheses

**Fig 1:**
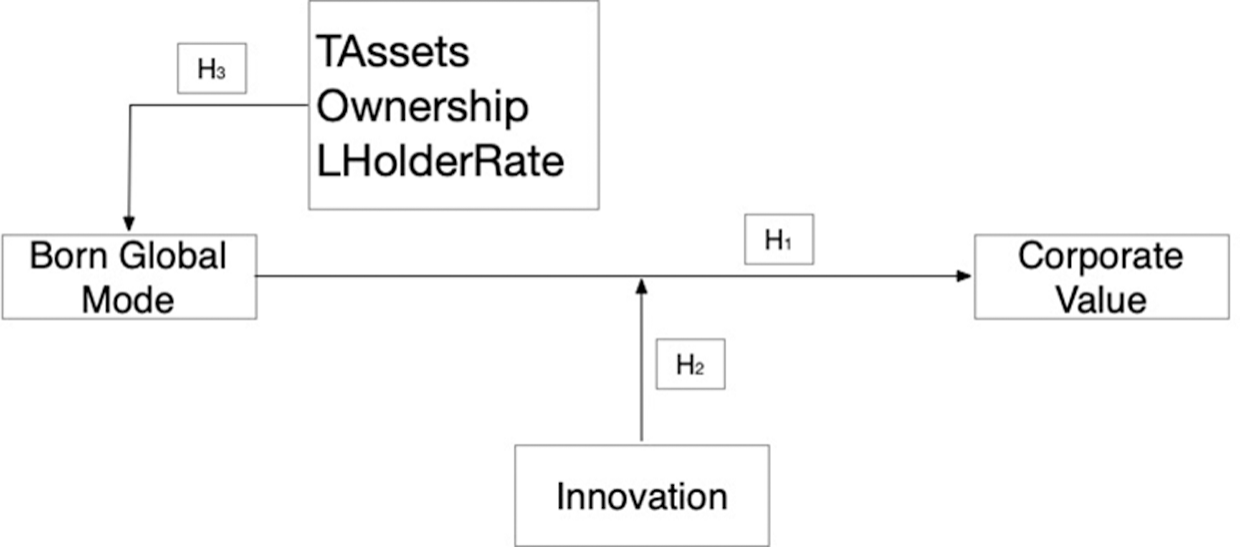
Conceptual framework. Note: Full variable definitions appear in S1 Appendix A.

According to existing research, the BG mode often brings positive financial performance and contributes to the growth of corporate value [22,53]. The innovation of BG firms may be one of the channels that drives corporate value [8]. The assets and equity of the company would also be determining factors in becoming a BG firm [18,61]. Therefore, as shown in Fig 1, Hypothesis 1 tests the effect of the BG mode on corporate value. Hypothesis 2 investigates the moderating effect of innovation on the relationship between the BG mode and corporate value. Hypothesis 3 discusses the determinant factors for selecting the BG mode, including total assets, ownership, and the rate of the largest shareholder.

#### 2.3.1. The effect of the BG mode.

Previous studies have examined the various effects of the BG mode on firm sales, firm size, and firm performance in both developed and developing countries including the company’s equity, growth, export volume, innovation, and corporate value. Ferguson, Henrekson [19] employed extensive empirical data to examine the long-term performance of BG firms in Sweden, challenging the widely held hypotheses in the extant literature about the inherent superiority of BG firms. It indicated that the implementation of BG strategies did not necessarily promote firm growth and emphasized the critical importance of accurately identifying BG firms in empirical analyzes. Kim, Lee [18] analyzed the differences of financial characteristics between BG and non-BG firms in South Korea, which prove that BG companies can make efficient use of assets and equity, but their profitability and financial stability are not good.

The impact of the BG mode on corporate value is the main interest of this study. There are few discussions on whether and how the selection of the BG mode has a positive relationship with corporate value, especially in China. Some studies only discuss the relationship between the BG mode and firm financial performance. For example, using the SMEs of BG in China, Zhou, Wu [53] found the mediating role of family-based social networks in the relationship between inward and outward internationalization and firm performance. She, Yu [48] confirmed that Chinese BG companies are positively related to enterprise performance, but not to enterprise size. Compared to global firms, Gull, Asghar [62] found that the entrepreneurial orientation of BG firms led to innovation and dynamic organizational capability, as well as improved the international performance and development of enterprises.

However, there is still a lack of consensus on the positive relationship between the BG mode and Chinese corporate value as well as the theoretical mechanism of the positive impact of the BG mode on Chinese corporate value. In the development situation of Chinese enterprises, based on the resource-based view, enterprises need to use limited resources to build sustainable competitive advantages [63,64]. Therefore, for enterprises, timely and efficient internationalization contributes to their operation, performance, and growth. Therefore, we developed the first hypothesis.

**Hypothesis 1.** *The BG mode is positively related to corporate value.*

#### 2.3.2. Moderating effect of innovation.

Innovation is the core of an enterprise. Without innovation, enterprises will lose their core competitiveness. The development of an enterprise cannot be separated from the support of innovation and innovation depends on the development of an enterprise. Innovation determines the direction, scale, and speed of a company’s development [65]. Innovation, R&D, knowledge development, and capabilities leveraging also play important roles in the internationalization of BG firms [8].

Some scholars have studied the role of innovation in BG companies, covering both developed and developing countries. For example, based on the study of Italian brands, the role of digital innovation in the transformation of BG digital enterprise into an enterprise brand was found and provided imperatives to transform into a competitive enterprise brand [52]. Using the structural equation model, Karatepe, Mithat Uner [66] found that the customer orientation of Turkish BG firms indirectly affects the innovation capability through the mediating effect of technical capability, but does not support the mediating effect of relationship quality and relationship information process. Knight and Liesch [67] proposed that the potential research direction of the BG mode is the impact of globalization and advanced technology on the performance of BG firms.

In emerging markets like China, startups generally lack innovative resources and talents in earlier times [68]. Although excellent enterprises trend towards technological innovation after owning capital, they lack patience for the long-term development of new technology [69]. In particular, some of Chinese “unicorn” enterprises have problems such as industrial bubbles, financing difficulties, high costs, and dispersed equity in their R&D investment [70]. Therefore, it is not clear whether innovation has a negative impact on the development of BG firms. For example, Wu [71] suggested that strong technical capability would inhibit the inverted U-shaped relationship between cooperative competition and product innovation performance. Kobarg, Stumpf-Wollersheim [72] also believes that the absorptive capacity of internal R&D negatively moderates this relationship. Kraft and Czarnitzki [73] argued that too much innovation is risky and reduces the value of the company. Although it is well known that the level of innovation will affect the value of enterprises, it is still unknown how Chinese startups adjust the positive relationship between the BG mode and enterprise value. We developed the second hypothesis as follows.

**Hypothesis 2.** *Innovation moderates the relationship between the BG mode and corporate value.*

#### 2.3.3. The determinant factors of selecting the BG mode.

Scholars discussed the determinant factors for companies to choose the BG mode. The success of most firms lies in developing the right business strategy [74]. Only by adopting a management mode and operating mode suitable for the enterprise can the corporate value continue to increase [75]. Ferguson, Henrekson [19] found that employees, sales, and value added are determinant factors of the BG mode among Swedish firms; however, these factors did not demonstrate a persistent and statistically significant advantage. Therefore, it is crucial to explore the company’s own characteristics. We propose the perspective of corporate capital and equity to analyze the determining factors for firms to choose the BG mode.

From the perspective of a firm’s capital, companies that dare to start international business in the early stages of establishment generally maintain a high level of total assets. Stronger capital helps SMEs to expand into the international market earlier [76]. However, Freeman, Edwards [77] studied the main constraints faced by smaller BG firms: lack of economies of scale, lack of resources (capital and knowledge), and unwillingness to take risks. Regarding the ownership type of enterprises, SOEs are generally less likely to try risky businesses, while POEs or FOEs are generally more keen to explore international markets [78]. Especially for companies with foreign investment background, it is easier to carry out early international business with foreign market resources. For the shareholding ratio of the largest shareholder, reducing the shareholding ratio of the largest shareholder helps reduce corporate financing costs [79]. Especially for start-up companies, if the ownership is too concentrated, it will infringe on the interests of small and medium-sized shareholders and affect investors’ confidence in the company [80,81]. Therefore, companies often have decision-making risks due to a lack of diversity [82].

Hence, corporate risks require entrepreneurs to adopt corresponding corporate strategies in time. To balance risks and returns, entrepreneurs usually choose internationalization [83,84]. For example, by considering the impact of expectation on internationalization decisions, Wadeson [85] found that when BG firms limit their negative risks by adopting progressive commitments, the impact of risk and expectation is consistent. However, other factors that affect expectation can change the balance of incentives to earlier and faster internationalization. Therefore, enterprises choose the right time to enter the BG mode by observing their own conditions. Therefore, we proposed Hypothesis 3 that BG firms are affected by their total assets, ownership, and the rate of the largest shareholder.

**Hypothesis 3.** *BG mode firms are influenced by their total assets, ownership, and the rate of the largest shareholder.*

## 3. Data and variables

### 3.1. Samples and data

We collected annual data from 2,064 firms listed on the Shanghai Stock Exchange and the Shenzhen Stock Exchange from 2007 to 2021 as an initial sample set. All financial data was obtained from the CSMAR database. We further examined SMEs listed on the “SME board” and the “Growth enterprise board” of the Shanghai Stock Exchange and Shenzhen Stock Exchange. By analyzing the data of Chinese small and medium enterprises, we extracted the financial characteristics of BG firms for discussion.

For data cleaning, this study carried out the following process. 1) Delete all financial companies. Because the financial data of financial companies does not truly reflect the financial condition of their enterprises, their financial information is significantly different from that of other companies. 2) Delete companies labeled “ST” (Special Treatment). ST refers to the two consecutive years of operating losses of domestic listed companies and warned of the risk of delisting. 3) Delete companies with significant outliers or missing key variables that violate accounting principles and common sense. 4) To reduce the influence of outliers, all continuous variables were winsorized at the 1st and 99th percentile. After data cleaning, the final sample of this study included 2,064 non-financial listed companies. Table 1 briefly describes the sample information.

**Table 1:** Descriptive statistics.

Variable	Obs	Mean	Std. Dev.	Min	Max
**Tobin’s Q**	7082	2.363	1.332	1.053	8.52
**BG**	20640	.017	.131	0	1
**Innovation**	2027	.36	.222	.078	1.133
**Age**	11540	6.951	8.488	0	27.25
**Ownership**	6502	1.971	.332	1	3
**lnTAssets**	7334	21.316	.844	19.612	23.763
**TATOR**	11451	.51	.353	.004	1.763
**TAGRate**	7384	.4	.697	−.295	3.775
**lnGRevenue**	7376	20.402	.992	18.138	23.351
**PERatio**	6431	85.845	110.557	13.936	783.159
**TLeverage**	11001	1.205	.661	.402	5.756
**LHolderRate**	6912	30.98	13.298	2.79	96.5

Note: The sample comprised 2,064 firms from 2007 to 2021. The main variables were winsorized at the 1st and 99th percentiles. Full variable definitions appear in S1 Appendix A.

### 3.2. Dependent variable

The dependent variable is the company value, which is usually represented by Tobin’s Q. Tobin’s Q calculates the ratio of the market value of stocks, the sum of the value of liabilities and the book value of assets at the end of the year. Tobin’s Q reflects the ability of corporate value to increase in a future period of time. In domestic and foreign studies, Tobin’s Q is used as a measure of corporate value. The greater the value of Tobin’s Q, the higher the corporate value. Because Tobin’s Q can accurately measure corporate value, this study selected Tobin’s Q as the measurement index of corporate value.

### 3.3. Independent variable

With regard to the independent variables in this study, a dummy variable named “born global” was created to identify BG. The firm is given a value of 1 if it meets the BG definition, otherwise the value is 0. Regarding the variable definition of BG, related studies are slightly different in different countries. Some studies define BG as a startup that exports at least 25% of its sales within three years after founding, as this is the most common definition in the literature. This definition was derived from Moen and Servais [86], and Knight and Cavusgil [8]. A few studies on Swedish companies used the 50% export threshold for robustness tests [19]. Other studies adopted the definition of BG as a company that has at least 10% of its sales in foreign markets who started international business within 3 years of inception [48,87]. For Korean companies, Kim, Lee [18] defined BG as a company that recorded overseas sales for the first time within three years of its inception.

However, it is difficult to for Chinese SMEs to achieve a high proportion of exports within a short period of time. Thus, the criteria used by the authors of this study to select BG firms are: (1) the maximum time from inception to starting international activities should no more than 3 years and (2) the share of foreign sales as a percentage of total sales should be above 10%. This is also the same as the definition of Chinese BG firms in the paper of She, Yu [48].

### 3.4. Moderating variable

The moderating variable is innovation. Innovation is usually measured by the number of patents issued in a year. However, there are many ways to measure innovation, such as the number of patents, R&D investment, the number of new products, etc. The relevant literature includes both continuous variables from databases and dummy variables from questionnaires. For example, Tang [88] used the number of patents applications as the level of innovation and the number of patent for invention to measure the quality of innovation. Additionally, the innovation variable defined by Altomonte, Gamba [89] is a dummy variable, taking R&D activities (in-house or outsourced) as measures of innovation. García-Quevedo, Segarra-Blasco [90] adopted the form of a questionnaire and defined a dummy variable as whether there are innovative R&D activities.

Since the characteristics of innovation are intangible, quantitative analysis is more complicated [88,91]. Therefore, we tried our best to choose quantitative indicators that can be statistically analyzed, such as the number of patents, R&D expenses, and R&D personnel. And, because these data are basically disclosed by enterprises, they are universal and consistent. We refer to the measurement method of Lu, Meng [70] and use the composite index to measure the innovation index. We use the following indicators to measure corporate innovation.

(1) Innovation input: the amount invested in R&D/ gross revenue
(2) Innovation output: total number of patents obtained by the enterprise for every year/ the maximum value of the year
(3) Innovation scale: the number of R&D personnel/ the number of employees of a listed firm

### 3.5. Control variables

This study controlled firm characteristics, ﬁrm growth, and firm profitability. For instance, we used ﬁrm age (Age), ownership type of enterprise (Ownership), total assets (TAssets), total assets turnover (TATOR), total assets growth rate (TAGRate), gross revenue (GRevenue), price earnings ratio (PERatio) and total leverage (TLeverage) as control variables. S1 Appendix A provides specific definitions for all variables.

## 4. Methodology

### 4.1. Pooled OLS models

In this study, we applied financial data to test the above hypotheses by a pooled OLS and panel models. First, we used the pooled OLS model to preliminarily analyze the relationship between the BG mode and corporate value. Then, by adding control variables and fixed effects of year and industry, we further regressed the model to minimize the biased estimation caused by endogenous problems. We proved the influence of the BG mode on corporate value by the pooled OLS model. In order to prove the moderator effect of innovation, we also examined the regression of the cross-term for BG and innovation.

The feature of panel data is that each individual has multi-period observations, which may introduce some problems to the regression. For example, the regression of data may have individual characteristics that cannot be observed (i.e., against exogeneity) and the disturbance terms of each individual in different periods may also be correlated (i.e., against homoscedasticity), so the estimated regression of panel data requires some special processing. We first used the pooled OLS model to estimate the fixed and random effects of the data. The purpose is to obtain the most basic results by the ordinary least squares method. Pooled OLS estimates take into account intra-group deviations (i.e., changes of each individual at different times) and inter-group deviations (i.e., differences between different individuals) with equal weights. The fixed effects model only considers intra-group deviations, so the influence of individual traits that do not change over time upon regression can be eliminated. The random effects model is equivalent to generalized least squares (GLS) regression. The estimated results are the weight of the intra-group estimator and the inter-group estimator, with the weight of the inter-group estimator between 0 and 1 (i.e., between the pooled OLS and FE estimated results). Therefore, we showed the effect of the fixed effect (FE) and random effect (RE) models on the financial data under the pooled OLS model.

The regression model is as follows. The model was used to test Hypothesis 1. Equation (1), where Tobin’s Q represents corporate value and BG is a dummy variable based on the conditions defined by the authors, was used to assess whether the BG mode influences the corporate value (i.e., Hypothesis 1). Here, (i) represents the enterprise i, (t) represents the time dimension (years), α0 is a constant, α1, α2, α3, and α4 are the regression coefficients, and ε is the stochastic term.


$$\begin{array}{c} Tobin^{\prime }s\;Q_{it}=\alpha_{0}+\alpha_{1}BG+\alpha_{2}{Innovation}_{it}+\\ \alpha_{3}{Controls}_{it}+\varepsilon \end{array}$$ (1)

$$\begin{array}{c} Tobin^{\prime }s\;Q_{it}=\alpha_{0}+\alpha_{1}BG+\alpha_{2}{Innovation}_{it}+\alpha_{3}BG*{Innovation}_{it}+\\ \alpha_{4}{Controls}_{it}+\varepsilon \end{array}$$ (2)

If α1 is significantly larger than 0, the hypothesis that the BG mode has positive marginal effects on corporate value was confirmed. We further explored the marginal effects of the BG mode on corporate value after adding moderator variable and control variables by Equation (2). The moderator variable innovation was added to evaluate whether innovation plays a moderating role between the BG mode and corporate value (i.e., Hypothesis 2). Together with the all variables, it was used to comprehensively test the influence of the BG mode on corporate value.

### 4.2. Panel models

We used the financial data of listed companies to further analyze the problems of enterprises by the panel regression method. By comparing the results of random effects and fixed effects, we found further evidence to support Hypothesis 1 and Hypothesis 2. The two methods of FE and RE are presented for the following reasons. The difference between the FE model and the RE model is that the missing individual characteristic variables cannot be determined whether they are explanatory variables or random error terms. The fixed effects model considers individual characteristic variables as explanatory variables, while the random effects model considers individual characteristic variables as random error terms. Because of this, the explanatory variables in the FE model can be related to individual characteristic variables, but not in the RE model. However, Sheytanova [92] confirmed that if the test is conducted according to the asymptotic critical values of the Hausman test, the null hypothesis will be over-rejected. Some scholars also argued that even if the Hausman test is passed, it does not mean that the hypothesis condition (H0) of the RE model is completely satisfied, but it is just a probability problem [93]. But, since the FE model is completely unaffected by this hypothesis, it is unbiased whether H0 is true or not. Of course, the cost of using the FE model is an inaccurate estimate. In short, the results may not be significant for the FE model, but significant for the RE model.

The panel regression model is the same as Equations (1 and 2). Tobin’s Q still represents the corporate value and BG represents BG firms defined by the author. (i) represents enterprise i, (t) represents the time dimension (year), α0 is a constant, α1, α2, α3, and α4 are regression coefficients, and ε is the stochastic term. If α1 is greater than 0, it is again confirmed that the BG mode has a positive effect on corporate value (i.e., Hypothesis 1). Then, the moderating variable “innovation” is added to explore whether innovation moderates the relationship between the BG mode and corporate value (i.e., hypothesis 2). After all variables are added, the influence of the BG mode on corporate value is proved.

### 4.3. Logistic models

Linear regression is a very important regression method, but it is only applicable to the case that the dependent variable is continuous variable. If the dependent variable is a categorical variable, logistic regression is required. Although both logistic regression and multiple linear regression are generalized linear models, the dependent variables are binomial distribution and continuous variables, respectively. Moreover, logistic regression can be divided into three categories. One is binary logistic regression, which is called binomial logistic regression. One is logistic regression with disordered multiple categories as the dependent variable, which is named multivariate logistic regression. One is logistic regression where the dependent variable is an ordered multiple classification, which is called cumulative logistic regression or sequential logistic regression. The explanatory variable of this model is BG, which is a binary variable. As a result, we adopted binomial logistic regression.

According to the characteristics of BG firms, we further distinguished their main characteristics. The total assets, ownership, and the rate of largest shareholder of firms were selected as the key factors to determine whether enterprises select the BG mode. We used logistic models to estimate the characteristic of BG firms. Equation (3) of this model is as follows. BG is a dummy variable based on the conditions defined by the author (i.e., BG firms). Moreover, (i) represents the enterprise i, (t) represents the time dimension (year), β0 is a constant, β1, β2, and β3 are the regression coefficients, and ε is the stochastic term.


$$\begin{array}{c} {BG}_{i}=\beta_{0}+\beta_{1}{TAssets}_{it}+\beta_{2}{Ownership}_{it}+\beta_{3}{LHolderRate}_{it\;}+\varepsilon \end{array}$$ (3)

$$\begin{array}{c} {BG}_{i}=\beta_{0}+\beta_{1}{TAssets}_{it}+\beta_{2}{Ownership}_{it}+\beta_{3}{LHolderRate}_{it\;}\\ +\beta_{4}{Controls}_{it}+\varepsilon \end{array}$$ (4)

If the regression coefficients β1, β2, and β3 can be significant at 1%, it is confirmed that the total assets, ownership, and the rate of largest shareholder are the influencing factors for the enterprise to enter the BG mode. In addition, we also added control variables to compare the marginal effects of the BG mode on the total assets, ownership, and the rate of largest shareholder of firms (i.e., Equation (4)). The RE model and FE model were used to further comprehensively test the relationship between the BG model and the total assets, ownership, and the rate of largest shareholder of firms.

## 5. Empirical results

### 5.1. Descriptive statistics

Table 1 reports basic statistics to depict the data for comprehensive understanding. Table 1 shows that the main indicators are quite different among Chinese-listed ﬁrms, including Tobin’s Q, BG, innovation and control variables. The natural logarithm of Tobin’s Q ranged from 1.053 to 8.52 across ﬁrms and the mean was 2.363. A mean value greater than 1 indicates that the market value of most selected ﬁrms is higher than the replacement costs of their assets. The mean value of innovation is 0.36, indicating that the innovation intensity of SMEs is not enough. Additionally, the average shareholding ratio of the largest shareholder is about 30%, indicating that the ownership of most SMEs is relatively concentrated.

BG are dummy variables (i.e., 0 or 1) and the mean is only 0.017, indicating that there are a few BG firms in China that meet the definition of this paper. Ownership type is a dummy variable, where SOEs are valued as 1, POEs valued as 2, and FOEs valued as 3. When the mean of ownership is 1.971, it indicates that non-SOEs make up the majority of the sample. The average age of the selected firms was 6.951 years old, indicating that many firms had been established for a relatively short time. Therefore, these samples meet the requirements of the study. The average of the firm’s total asset turnover ratio is higher than 50%, indicating that the most of the company’s financial position is sound.

### 5.2. Baseline regression of pooled OLS

Table 2 shows the main results of the empirical analysis. Each column shows the result of pooled OLS regression and is analyzed with the random and fixed effects model. As shown in Table 2, the estimation result of columns (1) and (2) is the regression result of Equation (1). Columns (1) and (2) show that the BG coefficient, α1, values are 0.256 and 0.271. Columns (3) and (4) are the estimated results of Equation (2), which are the regression results after adding the moderator variable and control variables. Columns (3) and (4) show that the BG coefficient, α1, values are 0.529 and 0.585, which are still significant at the 1% level. This result proves that the BG mode has a positive effect on corporate value. Accordingly, Hypothesis 1 is confirmed.

**Table 2:** Pooled OLS models.

	(1)	(2)	(3)	(4)
	Model1	Model2	Model3	Model4
**BG**	0.256***	0.271***	0.529***	0.585***
	(0.07)	(0.08)	(0.19)	(0.14)
**Innovation**	0.126***	0.138***	0.124***	0.134***
	(0.02)	(0.02)	(0.02)	(0.02)
BG* **Innovation**			0.227	0.261***
			(0.14)	(0.10)
**Cons**	YES	YES	YES	YES
**Year_Indus**	NO	YES	NO	YES
**Adj. R** ^**2**^	0.228	0.275	0.229	0.276
**N**	1595	1595	1595	1595

Note: Regression results of Equations (1 and 2). The sample comprises 2,064 firms from 2007 to 2021. The results are from random effects and fixed effects regression with Tobin’s Q as a dependent variable. Standard errors are in parentheses. *, **, and *** denote the significance at the 10%, 5%, and 1% levels, respectively. Full variable definitions appear in S1 Appendix A.

After adding moderating variables, pooled OLS regression analysis of fixed and random effects proceeds as follows. Columns (3) and (4) are the estimated results after adding Innovation as a moderating variable to the model. The coefficient values, α2, of BG × Innovation are 0.227 and 0.261, which are all significant at the 1% level. The coefficient of column (4) is similar to that of column (3). Therefore, Innovation as a moderating variable has a significant strengthening effect on the relationship between the BG mode and Tobin’s Q. Thus, Hypothesis 2 is confirmed. Compared to the result of the pooled OLS models, the coefficients of BG are always positive. These results are consistent with She, Yu [48] who showed the BG mode has a positive effect on corporate value. As a result, Hypothesis 1 is proved. But compared to the model of She, Yu [48], we added more variables related to financial ratios to describe the financial status of firms in detail. At the same time, we also use a larger sample and extended the period of observing variables in order to more comprehensively and objectively analyze the long-term impact of the BG mode on corporate value.

### 5.3. Baseline regression of the panel model

For the robustness of the results, we utilized the panel model to regress the observations. We compared the effects of different models considering random effects and fixed effects on the results. Columns (1) and (2) in Table 3 are the estimated results of Equation (1). The BG coefficient, α1, values in columns (1) and (2) were 0.687 and 0.748. Columns (3) and (4) are the estimation results of Equation (2), which are regression results when Innovation is added into the model as a moderator variable. The coefficients, α2, of BG × Innovation are 0.286 and 0.297, both of which are significant at the 1% level.

**Table 3:** Panel models.

	(1)	(2)	(3)	(4)
	Model1	Model2	Model3	Model4
**BG**	0.687***	0.748**	0.456***	0.503***
	(0.24)	(0.37)	(0.14)	(0.16)
**Innovation**	0.743***	0.965***	0.102***	0.129***
	(0.16)	(0.22)	(0.02)	(0.03)
BG* **Innovation**			0.286***	0.297***
			(0.09)	(0.11)
**Cons**	YES	YES	YES	YES
**Year_Indus**	NO	YES	NO	YES
**N**	1595	1595	1379	1379

Note: Regression results of Equations (1 and 2). The sample comprises 2,064 firms from 2007 to 2021. The results are from random effects and fixed effects regression with Tobin’s Q as a dependent variable. The standard errors are in parentheses. *, **, and *** denote significance at the 10%, 5%, and 1% levels, respectively. Full variable definitions appear in S1 Appendix A.

According to the test of moderating effect, if the main effect coefficient, α1, is significantly positive and the cross-term coefficient is positive, it indicates that the moderating variable strengthens the relationship between the main effects. In other words, Table 3 shows that innovation significantly strengthens the relationship between the BG mode and Tobin’s Q. Therefore, Hypothesis 2 was confirmed. Compared with the pooled OLS regression results, the panel regression results better supported Hypotheses 1 and 2. Therefore, the results are consistent with the previous conclusion that the BG mode has positive effects on corporate value and innovation plays a positive moderating role. Compared with other studies, we add the comprehensive index of innovation as moderating variables in order to discover the influence mechanism between the BG mode and corporate value.

### 5.4. Logistic regression with BG firms

This study finds that companies that choose the BG mode are related to their total assets, ownership, and the rate of largest shareholder. Table 4 shows the main results of the empirical analysis. We use BG mode as the dependent variable to perform logistic regression for Equations (3 and 4). As shown in Table 4, the estimation results in columns (1) and (2) are the regression results of Equation (3). Column (1) shows that the Logistic regression of the total assets, ownership, and the rate of the largest shareholder, which are both significant at the 1% level. Column (2) is the estimated result of a fixed effect, which is the regression result after fixing the variables with year (Year) and industry (Indus). Column (2) shows that the coefficients of the independent variables are 0.927, 0.793, and −0.031, which are still significant at the 1% level. In addition, the results of columns (3) and (4) are estimated after adding control variables. All of the variables are significant at the 1% level. Accordingly, Hypothesis 3 is confirmed.

**Table 4:** Logistic regression with BG firms.

	(1)	(2)	(3)	(4)
	Model1	Model2	Model3	Model4
**TAssets**	0.398***	0.927***	0.338**	0.962***
	(0.08)	(0.11)	(0.14)	(0.19)
**Ownership**	0.757***	0.793***	1.023***	1.178***
	(0.22)	(0.23)	(0.25)	(0.25)
**LHolderRate**	−0.022***	−0.031***	−0.024***	−0.032***
	(0.01)	(0.01)	(0.01)	(0.01)
**Cons**	NO	NO	YES	YES
**Year_Indus**	NO	YES	NO	YES
**N**	6476	5566	5621	5063

Note: Logistic regression results of Equations (3 and 4). The sample comprises 2,064 firms from 2007 to 2021. The results are from random effects and fixed effects regression with BG as a dependent variable. The standard errors are in parentheses. *, **, and *** denote significance at the 10%, 5%, and 1% levels, respectively. Full variable definitions appear in S1 Appendix A.

The above results reflect the determinant factors of a firm’s choice of the BG mode. We compare the effects of total assets, ownership, and the rate of largest shareholder on the BG mode. According to the regression results, the higher the total assets, the more powerful the firm is to adopt the BG mode for development. For ownership, the results are positive and significant at the 1% level. This shows that the type of ownership does affect whether a firm chooses to enter the BG mode. The more the ownership structure tends to non-SOEs, the easier it is for a firm to choose the BG mode. There is a significant negative relationship between the rate of the largest shareholder and the BG mode. This indicates that reducing the equity ratio of major shareholders would help start-up companies reduce financing costs and help actively develop the BG mode. Therefore, Hypothesis 3 is confirmed. Compared to a study by She, Yu [48], we selected different determinant factors to analyze. We did not consider the region and firm size, but found the determinant factors from firm’s own characteristics. Different from research by Kim, Lee [18], we found the unique characteristics of BG firms not only from the financial perspective, but also from the equity perspective.

### 5.5. Results of Heckman two-stage

To solve the bias of sample selection, we utilize the Heckman two-stage method to avoid the endogeneity problem of the model [94]. Since the firms export of the database may be inaccurate or not disclosed, the estimates of BG based on the value may be biased. In addition, the dependent variable of corporate value may be omitted, which also causes the regression results to be biased. Therefore, we introduce an instrumental variable as an exclusive variable to avoid the multicollinearity problem in the regression results. We refer to the instrumental variable InDWP from the paper of Cai, Yun [95], that is, whether the workplace of independent directors is the same as the registered place of listed firms. When independent directors are in the same place as the company, their supervision may interfere with depend decision-making of entrepreneurs, which is not conducive to the effective implementation of the BG mode.

In the first stage, to design a probability model for firms to choose the BG mode, we first transform the dependent variable of corporate value into a binary variable, defined as Tobin’s Q_hat. Then, the mean of Tobin’s Q is used as the threshold. If it is greater than the mean, the assigned value is 1, otherwise it is 0. Then, according to the Probit model, the Inverse Mills Ration (IMR) of each sample is calculated. In the second stage, IMR is added as a control variable in Equation (6) and the logit model is used to estimate the regression parameters. The two-stage model is shown in Equations (5 and 6).


$$\begin{array}{c} Tobin^{\prime }s\;Q_{it}\_hat=\gamma_{0}+\gamma_{1}InDWP+\gamma_{2}BG+\gamma_{3}{Patents}_{it}+\\ \gamma_{4}{Controls}_{it}+\varepsilon \end{array}$$ (5)

$$\begin{array}{c} Tobin^{\prime }s\;Q_{it}=\alpha_{0}+\alpha_{1}BG+\alpha_{2}{Patents}_{it}+IMR\\ \alpha_{3}{Controls}_{it}+\varepsilon \end{array}$$ (6)

As shown in Table 5, column (1) shows the regression results of the first stage by Equation (5). The instrumental variables better identify exclusion restriction variables and it is observed that the effect of BG on corporate value has a level of significance. Moreover, independent directors are co-located with the company, which has a negative effect on the implementation of the BG mode, thereby affecting changes in the corporate value. Column (2) is the regression result of the second stage by Equation (6). The main effect of the BG mode and corporate value is also significant at the 5% level. However, the IMR is significant, indicating that the original model has serious sample selection bias. Therefore, the regression results obtained by using the Heckman two-stage method are robust and similar to the results reported in Table 1.

**Table 5:** Results of Heckman two-stage.

	(1)	(2)
	Stage 1	Stage 2
**InDWP**	−0.137*	
	(0.08)	
**BG**	1.968**	0.719**
	(0.96)	(0.32)
**Innovation**	1.278***	−0.100**
	(0.20)	(0.05)
BG*Innovation	1.456*	1.822***
	(0.84)	(0.64)
**Inverse Mills Ratio**		−0.415***
		(0.09)
**Cons**	YES	YES
**Year_Indus**	YES	YES
**Adj. R** ^**2**^		0.153
**N**	1368	457

Note: Heckman two-stage results of Equations (5 and 6). The sample comprises 2,064 firms from 2007 to 2021. The results are from two-way fixed effects regression with Tobin’s Q_hat and Tobin’s Q as a dependent variable, respectively. InDWP is an exclusive variable as an instrumental variable. The standard errors are in parentheses. *, **, and *** denote significance at the 10%, 5%, and 1% levels, respectively. Full variable definitions appear in S1 Appendix A.

### 5.6. Results of PSM

There may be endogeneity problems in the process of selecting BG firms, that is, whether a firm chooses the BG mode is likely to be affected by the characteristics of the sample. For example, firms with advanced technology are more likely to choose the BG mode and entrepreneurs with high educational levels are also more likely to choose the BG mode. Therefore, it is required to estimate the extent to which the change in corporate value is caused by the choice of the BG mode. We divide the firms that chose the BG mode into two groups, treatment and control groups. We find individuals in the control group who are similar to the treatment group for matching. In this case, we can eliminate the impact of corporate characteristics on corporate value, so that the selection of the BG mode is as random as possible. First, we randomly sort the samples and allow for matching with ties. Due to the limited number of samples, we also chose matching with replacement. To minimize the mean square error (MSE), we use one-to-four matching based on the work of Abadie, Drukker [96]. Finally, we select k-nearest neighbor matching with a caliper of 0.01 and test that there is no significant difference between the treatment and control groups after matching. That is to prove that the assumption of equilibrium is satisfied.

**Fig 2:**
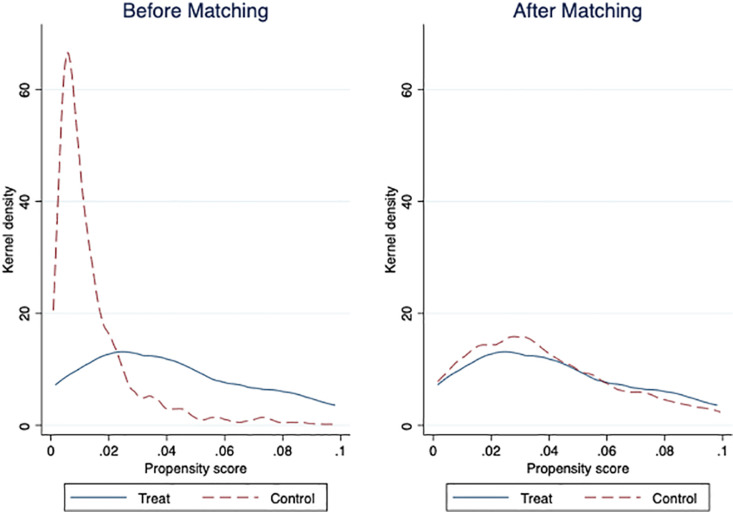
Kernel density of propensity scores before and after matching. Notes: This figure displays kernel density estimates of propensity scores before and after matching. The sample comprises 2,064 firms from 2007 to 2021. Using k-nearest neighbor matching with a caliper of 0.01, we partitioned firms into treatment and control groups based on characteristics similarity. The blue solid line represents the treatment group; the red dotted line indicates the control group.

As shown in Fig 2, by comparing the kernel density estimation plot of coefficients before and after matching, we find that the two groups of samples have significant imbalances in some features before matching. After matching, the kernel density function plots of the two groups of samples become closer. Moreover, the peak values of the propensity scores tend to be consistent and the shapes of the kernel density functions are also more similar. This shows that our matching process effectively balanced the differences in the key characteristics between the two groups of samples, making the two groups of samples more comparable. That is to say, the matching results obtained by the matching method are more reliable.

After we match the samples of the treatment and the control group by k-nearest neighbor matching, we compared the difference of the corporate value between enterprises that choose the BG mode (the treatment group) and do not choose the BG mode (the control group) under the same conditions. We verify that there is a significant causal relationship between whether to choose the BG mode and corporate value. The specific regression results are as follows. Table 6 shows the estimated results of Equation (1) after propensity score matching. Columns (1) and (2) are the results of the pooled OLS model, and columns (3) and (4) are the results of the panel model. In addition, columns (2) and (4) are the fixed effect regression with years and industry level. All regression models are clustered for individuals to avoid biased estimates of the results. As shown in Table 6, we match 113 BG firms with 113 no-BG firms and most of the main effects are significant at the 5% level. The results based on matched samples are similar to those reported previously in Tables 3 and 4. The results further prove that the choice of the BG mode is conducive to the improvement of corporate value. Therefore, Hypothesis 1 is strongly supported.

**Table 6:** Results of PSM.

	Pooled OLS		Panel	
	Model1	Model2	Model3	Model4
**BG**	0.566***	0.576**	0.387**	0.365
	(0.20)	(0.24)	(0.18)	(0.24)
**Innovation**	0.427**	0.436*	0.009	0.007
	(0.19)	(0.22)	(0.24)	(0.09)
BG*Innovation	0.263**	0.280*	0.161	0.159
	(0.12)	(0.16)	(0.12)	(0.17)
**Cons**	YES	YES	YES	YES
**Cluster**	YES	YES	YES	YES
**Year_Indus**	NO	YES	NO	YES
**Adj. R** ^**2**^	0.309	0.261		
**N**	113	113	113	113

Note: PSM regression results of Equation (2). The sample comprises 2,064 firms from 2007 to 2021. The results are from random effects and fixed effects regression with Tobin’s Q as a dependent variable. The firms with similar characteristics were divided into treatment and control groups by k-nearest neighbor matching with a caliper of 0.01. Firm-clustered standard errors are in parentheses. *, **, and *** denote significance at the 10%, 5%, and 1% levels, respectively. Full variable definitions appear in S1 Appendix A.

### 5.7. Results of heterogeneity analysis

Next, we compare the differences of the corporate value with the BG mode according to the ownership of the enterprise. In Table 7, columns (1), (2), and (3) are pooled OLS analysis results of SOEs, POEs, and FOEs, respectively. The coefficient of BG only shows significant effects among POEs and FOEs, indicating that the implementation of the BG mode by SOEs may not increase corporate value. The coefficients in Columns (2) and (3) are 0.689 and 0.467, respectively, which shows that the implementation of the BG mode in POEs contributes more to the growth of corporate value. By observing the coefficient related to innovation, we find that among the effects of innovation on corporate value, FOEs have the strongest effect, while the moderating effect is indeed the weakest. This shows that FOEs win the market through new technological innovations. However, due to early mastery of advanced technology, the moderating effect of innovation on its corporate value tends to weaken.

**Table 7:** Results of heterogeneity analysis sorted by ownership type.

	(1)	(2)	(3)
	SOEs	POEs	FOEs
**BG**	0.255	0.689***	0.467***
	(0.23)	(0.14)	(0.10)
**Innovation**	0.130**	0.097***	0.416***
	(0.06)	(0.02)	(0.09)
BG*Innovation	0.334	0.303***	0.000
	(0.22)	(0.10)	(.)
**Cons**	YES	YES	YES
**Adj. R** ^**2**^	0.236	0.226	0.425
**N**	131	1379	85

Note: Regression results of Equation (2) specifically for different levels of firms. Firms are classified as SOEs, POEs, and FOEs based on ownership type. The sample comprises 2,064 firms from 2007 to 2021. The results are from random effects regression with Tobin’s Q as a dependent variable. Standard errors are in parentheses. *, **, and *** denote significance at the 10%, 5%, and 1% levels, respectively. Full variable definitions appear in S1 Appendix A.

As shown in Table 8, we conducted group regression according to the traditional geographical divisions of China. Columns (1), (2), and (3) represent the western region, central region, and eastern region, respectively. We found similar results, that is, the BG mode has a positive effect on corporate value. In Column (3), the coefficient of BG is 0.541 and is significant at the 1% level. This shows that companies in eastern China will have a more significant increase in corporate value by adopting the BG mode. Moreover, the moderating effect of innovation is also significant in the regression estimation of the eastern region. Therefore, the results for firms in the eastern region support Hypotheses 1 and 2.

**Table 8:** Results of heterogeneity analysis sorted by region.

	(1)	(2)	(3)
	Western region	Central region	Eastern region
**BG**	0.000	0.805	0.541***
	(.)	(1.47)	(0.15)
**Innovation**	0.118	0.028	0.171***
	(0.10)	(0.05)	(0.02)
BG*Innovation	0.000	1.108	0.220**
	(.)	(1.34)	(0.09)
**Cons**	YES	YES	YES
**Year_Indus**	YES	YES	YES
**Adj. R** ^**2**^	0.374	0.365	0.282
**N**	103	228	1264

Note: Regression results of Equation (2) specifically for different levels of firms. Firms are classified as the western region, the Central region, and the eastern region based on region. The sample comprises 2,064 firms from 2007 to 2021. The results are from fixed effects regression with Tobin’s Q as a dependent variable. Standard errors are in parentheses. *, **, and *** denote significance at the 10%, 5%, and 1% levels, respectively. Full variable definitions appear in S1 Appendix A.

## 6. Discussion, implications, and conclusions

### 6.1. Discussion

BG firms, which rapidly enter international markets during their initial establishment phase, have achieved accelerated growth by strategically leveraging globalized resources. The emergence of BG firms has fundamentally broken the conventional “domestic-first, international-later” development model, with their strategic significance becoming increasingly prominent in contemporary economic environment. The development of BG firms has not only reconfigured the division of labor pattern within global value chains and enhanced the efficiency of cross-border innovation diffusion but also established novel competitive ecosystems in global markets and catalyzed iterative advancements in international governance frameworks.

In response to increasingly volatile market conditions and diversified consumer demands, BG firms must continuously strengthen their innovation capabilities to sustain competitive advantages in dynamic environments. However, excessive innovation may introduce operational uncertainties, a phenomenon that substantiates the critical role of dynamic capabilities theory in moderating the relationship between BG mode and corporate value within innovation intensity, while theoretically expanding the applicability boundaries of dynamic capabilities. Concurrently, the financial status and ownership structure of SMEs—shaped by their unique resource endowments—exert profound influences on BG mode selection. The level of enterprises’ resources not only determine their international expansion capacities but also shape their dependence on resources and risk propensity in cross-border investments. Particularly in navigating complex international markets, the observed variations in BG implementation across firms with heterogeneous ownership types provide critical micro-foundational insights. Furthermore, the interplay between financial status and ownership structure contributes to developing a more comprehensive theoretical framework for understanding the evolution of resource-based view within BG mode.

Based on the development of SMEs in emerging markets, this study will explore the influence of Chinese SMEs choosing the BG mode on corporate value from the perspective of enterprise innovation. Especially in recent years, with the rise of emerging market economies, more and more enterprises from developing countries tend to explore overseas markets. Through low cost, high efficiency, and technology transfer, many small but advanced enterprises gradually gain market share advantage. It has not only resulted in huge profits for companies, but also developed self-own brands from emerging economies.

Therefore, we suggested that governments and regulators in emerging markets attach importance to the development of SMEs and actively improve their investment and financing channels so that these enterprises can compete in overseas markets smoothly. Meanwhile, we also suggest that business managers effectively evaluate the development stage of the enterprise and timely implement the corporate strategy of the BG mode. This not only contributes to the rapid growth of corporate value, but can also create the international brand effect as early as possible. In brief, enterprise internationalization can improve corporate performance and change the level of corporate valuation in the long run.

### 6.2. Conclusions

This paper analyzes the non-financial SMEs listed in China’s stock market from 2007 to 2021. Based on the pooled OLS and panel models, we studied the change of corporate value when enterprises choose the BG mode. In summary, the results presented in this paper provide supporting evidence for the positive impact of the BG mode on the value of Chinese enterprises and its potential mechanism. In addition, we also found that Chinese SMEs should focus on the positive moderating effect of innovation on corporate value if they choose the BG mode. In addition, observing variables such as the total assets, ownership, and the rate of largest shareholder can help managers determine whether an enterprise chooses the BG mode or when it enters the BG mode. The development of POEs and FOEs choosing the BG mode would help increase corporate value. The adoption of the BG mode by firms in the eastern region significantly increases corporate value.

### 6.3. Implications and future research

The findings of the study have some implications for practitioners, investors, and policy makers. First of all, firms’ managers should actively choose the right time to start the process of internationalization. The evidence from the empirical analysis shows that the earlier an enterprise implements the internationalization strategy, the easier it is to improve company performance and value. However, while society and government encourage research and innovation, the managers must be clear-eyed about how much to invest in innovation. Excessive pursuit of innovation may affect the firm age and performance of BG firms. Start-ups can also use or customize existing technologies and launch new products into the domestic market by knowledge transfer [97].

Furthermore, it is imperative for investors and sophisticated corporate venture capital (CVC) entities to recognize the critical role of corporate internationalization strategies in enhancing enterprise market valuation. However, our analysis reveals that excessive emphasis on breakthrough technological innovation may engender strategic misalignment risks, particularly within developing economies. Institutional voids and underdeveloped innovation ecosystems in emerging markets often elevate the feasibility threshold for indigenous firms to achieve radical innovation outcomes [98,99]. Meanwhile, CVC entities should incorporate government industrial policy directives into their investment evaluation frameworks. Aligning with national innovation system trajectories through this policy-sensitive orientation enables strategic compliance with state-mandated technological priorities, thereby enhancing investment efficiency [69].

Finally, for national policy makers, we suggest that government should still encourage innovation and increase innovation subsidies in order to further improve the initiative of innovation. In particular, it is necessary to develop small but advanced enterprises like BG firms. In addition, policies should be set reasonable thresholds to apply to SMEs. The reason to stimulate corporate innovation is to realize the localization of all cutting-edge technologies, in order to cope with the blockade of foreign advanced technologies.

A limitation of this paper is that due to the lack of early data, the research on the BG mode has not been fully studied. In the future, if SMEs disclose more detailed financial data, it will provide strong support for research of BG. In addition, although this study uses a composite index to measure corporate innovation, it can only reflect the innovation behavior of enterprises to a certain extent. Although patents and R&D investment represent the innovation of enterprises, more indicators are needed to describe the innovation behavior. In the future, the innovation index can be measured from more dimensions, such as the number of new products, innovation performance, etc.

## Supporting information

S1 Appendix A : Table A1. Definitions of variables. (PDF)

S2 Data Comprehensive research dataset. (XLSX)
