# Supplementary material for: Innovation and valuation of Chinese born-global firms
Source: PLoS One. 2025 Jun 27;20(6):e0325214. doi: 10.1371/journal.pone.0325214 (PMC12204524; doi:10.1371/journal.pone.0325214)
Supplement: S1 Appendix — (PDF) [file pone.0325214.s001.pdf]

Appendix A:

Table A1. Definitions of variables

Variable type	Name of variable	Variable symbol	Variable definition
Dependent variable	Corporate value	Tobin's Q	The natural logarithm of the current Tobin's Q value of the firm
Independent variable	Born global	BG	The maximum time from inception to starting international activities should be no more than 3 years, and the share of foreign sales as a percentage of total sales should be above 10%
Moderator variable: Innovation	Input: R&D costs	RDCR	The amount invested in R&D / gross revenue
	Output: Patent	Patent	Total number of patents obtained by the enterprise for every year / the maximum value of the year
	Scale: R&D personnel	RDPR	The number of R&D personnel/ the number of employees of a listed firm
Instrumental variable	Workplace of independent directors	InDWP	Whether the workplace of independent directors is the same as the registered place of listed firms
Control variable	Firm age	Age	Date of establishment of firms
	Ownership type of enterprise	Ownership	The type of ownership: state-owned enterprises (1), private enterprises (2), foreign-owned enterprise (3)
	Total assets	TAssets	The natural logarithm of total assets of firms
	Total assets turnover	TATOR	The natural logarithm of net sales divide by total assets
	Total assets growth rate	TAGRate	The natural logarithm of total assets growth at the end of the year divide by total assets at the beginning of the year

Gross revenue	GRevenue	The natural logarithm of the total revenue gained from sales
Price earnings ratio	PERatio	The natural logarithm of the market value price per share divide by the company's earnings per share
Total leverage	TLeverage	Operating leverage ratio multiplied by financial leverage ratio
Largest holder rate	LHolderRate	The shareholding ratio of the largest shareholder
Type of industry	Indus	CSRC industry classification code from 2012

Source: Author.
